# Celastrol ameliorates osteoarthritis *via* regulating TLR2/NF-κB signaling pathway

**DOI:** 10.3389/fphar.2022.963506

**Published:** 2022-08-10

**Authors:** Guangxia Yang, Kai Wang, Hua Song, Rujie Zhu, Shuai Ding, Hui Yang, Jian Sun, Xin Wen, Lingyun Sun

**Affiliations:** ^1^ Nanjing Drum Tower Hospital, Clinical College of Traditional Chinese and Western Medicine, Nanjing University of Chinese Medicine, Nanjing, China; ^2^ Department of Rheumatology, Affiliated Hospital of Jiangnan University, Wuxi, Jiangsu Province, China; ^3^ Department of Rheumatology, Affiliated Huai’an No 1 People’s Hospital of Nanjing Medical University, Huaian, Jiangsu Province, China; ^4^ Department of Rheumatology and Immunology, The Affiliated Drum Tower Hospital of Nanjing University Medical School, Nanjing, China; ^5^ Department of Rheumatology and Immunology, Nanjing Drum Tower Hospital Clinical College of Nanjing Medical University, Nanjing, China

**Keywords:** osteoarthritis, celastrol, innate immunity, TLR2, NF-κB

## Abstract

**Objectives:** Osteoarthritis (OA) is a joint disease characterized by degeneration of joint cartilage and is a significant cause of severe joint pain, physical disability, and impaired quality of life in the aging population. Celastrol, a Chinese herbal medicine, has attracted wide interests because of its anti-inflammatory effects on a variety of diseases. This study aimed to investigate the effect of celastrol on OA as well as the mechanisms *in vivo* and *in vitro*.

**Methods:** A rat knee OA model was established using “medial collateral ligament transection (MCLT) + partial meniscectomy (pMMT)”. Eight weeks after surgery, the OA rats started to receive intra-articular injection of celastrol (1 mg/kg) once a week. Safranin O-fast green (S&F) and hematoxylin and eosin (H&E) staining were used to estimate histopathological changes. Micro-CT was used to evaluate bone volume of the subchondral bone of the knee joint. Chondrocytes were isolated from the knee cartilage of rats and OA patients. Enzyme linked immunosorbent assay (ELISA), Western Blot (WB), Polymerase Chain Reaction (PCR), and Immunohistochemistry (IHC) were used to detect the expression of inflammatory factors and stromal proteins, respectively.

**Results:** We found that celastrol treatment significantly delayed the progression of cartilage damage with a significant reduction in osteophyte formation and bone resorption in OA rat model. In IL-1β-stimulated rat chondrocytes, celastrol significantly suppressed the production of inflammatory factors such as cyclooxygenase-2 (COX2), interleukin-6 (IL-6), and prostaglandin E2 (PEG2), and reduced IL-1β-induced matrix degradation by down-regulating the expression of matrix metalloproteinase 13 (MMP13). In addition, we found that toll-like receptor 2 (TLR2) was up-regulated in OA patients and rat knee OA models, while celastrol inhibited TLR2 signal and its downstream nuclear factor-kappa B (NF-κB) phosphorylation.

**Conclusion:** In summary, celastrol may improve OA by inhibiting the TLR2/NF-κB signaling pathway, which provides innovative strategies for the treatment of OA.

## Introduction

Osteoarthritis (OA) is a chronic joint disorder, which has become a great burden on individuals affected, health care system and broader socio-economic costs (Martel-Pelletier et al., 2016; Hunter and Bierma-Zeinstra, 2019). The main symptoms of OA include joint pain, swelling, deformity and progressive joint activity disorder, which eventually leads to severe limitation of movement and decreased quality of life. Clinically, multiple joints can be involved in OA, especially knee joint, followed by hand and hip (Prieto-Alhambra et al., 2014; Turkiewicz et al., 2014). There are several risk factors that could lead to OA including heredity, environment, dislocation of the knee joint, biomechanical change of joint, overactivity, weight-bearing exercise, metabolic syndrome, and inflammation (Valdes and Spector, 2011; Berenbaum et al., 2018; Bortoluzzi et al., 2018; Cooper et al., 2018; Alberton et al., 2019). Accordingly, the pathogenesis of OA is polyfactorial, with age and obesity being the most influential factors (Hindy et al., 2019; Driban et al., 2020; Hart et al., 2020), both of which are associated with an abnormal inflammatory innate immune response that leads to OA progression (Kalaitzoglou et al., 2017).

Innate immunity participates in the development of OA by inducing a low-grade inflammatory response through damage-associated molecular patterns (DAMPs) and promoting synovitis and cartilage degradation through pattern-acting recognition receptors (Liu-Bryan, 2013). Accordingly, as the core class of pattern recognition receptors (PRRs) in the inflammation scenario, toll-like receptors (TLRs) recognize multiple DAMPs leading to the activation of inflammatory pathways such as nuclear factor-kappa B (NF-κB) and mitogen-activated protein kinase (MAPK) pathways, with subsequent release of cytokines, chemokines, and proteases (Miller et al., 2019). Release of inflammatory mediates, for example interleukin-6 (IL-6), tumor necrosis factor-α (TNF-α) and nitric oxide (NO), as well as chondrocytic mediators, including prostaglandin E2 (PGE2) and matrix metalloproteinases (MMPs), promoted chondrocyte apoptosis and extracellular matrix (ECM) degradation (Goldring and Goldring, 2007; Liu-Bryan and Terkeltaub, 2015). Notably, toll-like receptor 2 (TLR2) is considered necessary in the early stage of inflammation (Abdollahi-Roodsaz et al., 2008). In the progression of OA, DAMPs/TLR2 is a key pathway to the catabolism and anabolism of cartilage ECM(Kim et al., 2006; Hwang et al., 2015; Hwang et al., 2019). Several studies have shown that TLR2-mediated immune responses regulate cytokine gene expression by activating NF-κB, while inhibition of TLR-2/NF-κB signaling reduces the expression of multiple inflammatory cytokines (Arya et al., 2021; Lee et al., 2022). These findings enlighten us to examine whether TLR-2/NF-κB play a role in activation of the innate immune system by mediating cytokine release and ultimately delay OA progression.

The medical treatment of OA has been no substantial progress for a long time with given the major challenges such as repair and regeneration of cartilage or bone loss (Maqbool et al., 2021). Up to date, physical exercise and weight loss remain the cornerstones of OA treatment. In the initial stage of OA, non-steroidal anti-inflammatory drugs (NSAIDs) are widely used to relieve pain. Although symptoms can be alleviated, the progression of OA cannot be delayed. Short-term (6 weeks) treatment with 10 mg prednisolone is effective and safe for patients with sudden onset of hand OA (Kroon et al., 2019). However, long-term usageof glucocorticoids can generate numerous adverse effects. Moreover, oral drugs have non-negligible side effects, such as the potential risk of damaging digestive and cardiovascular system (McAlindon et al., 2014; Bindu et al., 2020).

Celastrol, an active compound extracted from root bark of Tripterygium wilfordii, mainly distributed and cultivated in Asian countries, showssignificant immunosuppressive properties (Zhang et al., 2021). The anti-inflammatory effect of celastrol has been well attested in autoimmune arthritis (An et al., 2020; Cascao et al., 2020). As a valuable inhibitor of NF-κB signaling pathway, celastrol shows anti-inflammatory and bone protective effects in the adjuvant-induced arthritis model (Cascao et al., 2020). Moreover, it has been found that celastrol plays a cardioprotective role in rheumatoid arthritis by inhibiting autophagy mediated by TLR2/HMGB1 signaling pathway (Lu et al., 2021). Compared with autoimmune arthritis, OA is mainly characterized by chronic, relatively low-grade inflammation. To date, few studies have examined the treatment of OA by celastrol comprehensively and thoroughly. Furthermore, whether celastrol can improve low-grade inflammation by inhibiting DAMPs-PRRs signaling has not been studied. Nor has the possible link between celastrol and chronic inflammation been explored in OA treatment.

This study aimed to evaluate the therapeutic effects of celastrol on OA and explore the molecular mechanism of celastrol in the treatment of OA.

## Methods

### Ethics statement

Human control samples were collected from amputees. Human OA cartilage samples were collected from OA patients during total knee arthroplasty. The protocols for collecting and analyzing human articular cartilage was approved by the Ethical Committee of Nanjing Drum Tower Hospital Clinical College of Traditional Chinese and Western Medicine (2020-156-01). All experimental procedures followed the guidelines of the Declaration of Helsinki (World Medical, 2013). All animal operation, treatment and post-processing were performed in strict accordance with the guidance of the Professional Animal Care and Use Committee of Nanjing Drum Tower Hospital Clinical College of Traditional Chinese and Western Medicine (2020AE02042).

### Rat model and treatment

**FIGURE 1:**
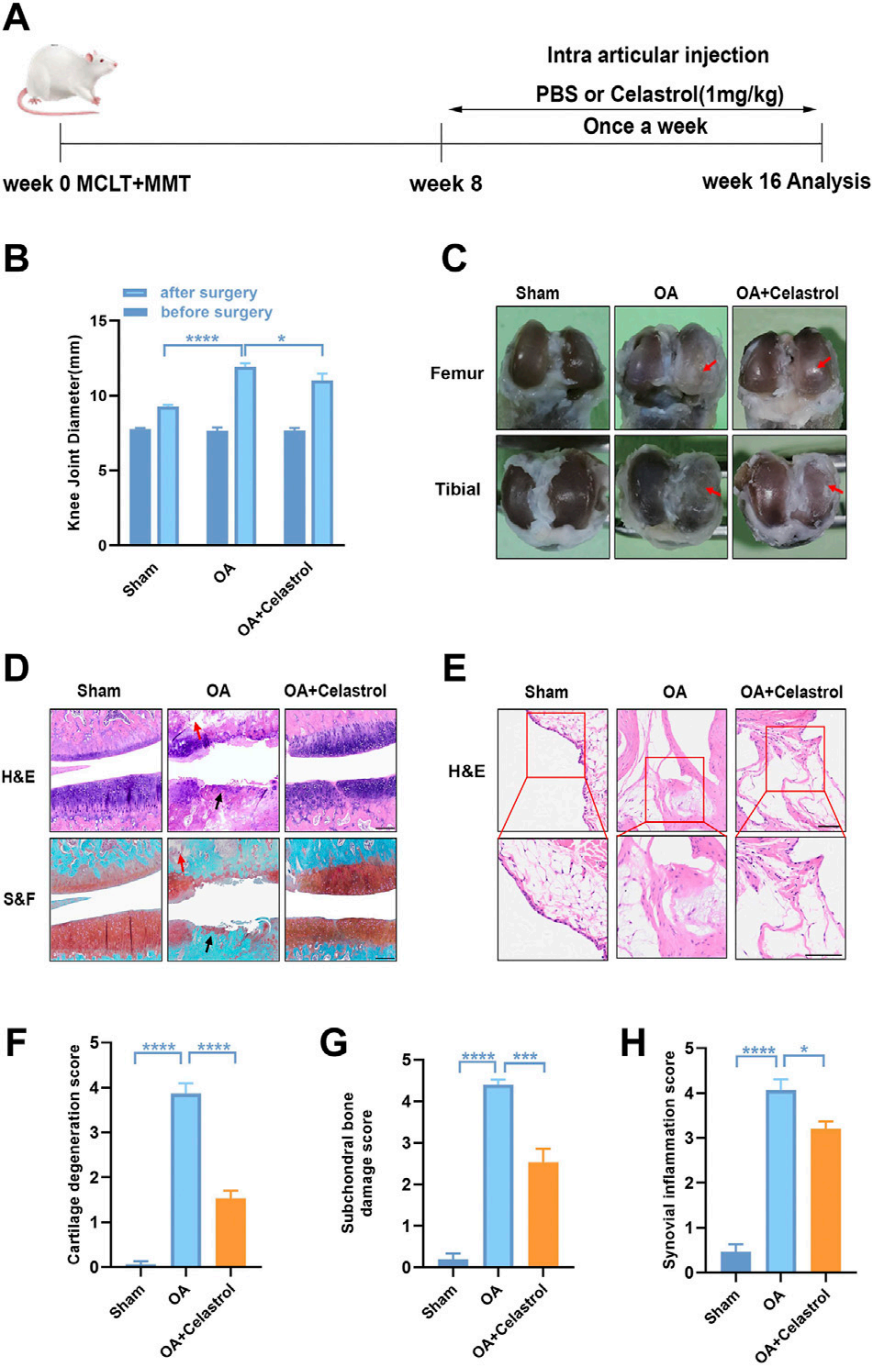
Celastrol alleviated the progression of OA in rat model. **(A)** Timeline for depicting overall the design of the study. **(B)** Knee joint diameter of the affected limbs of rats before surgery and 16 weeks after surgery; n = 6. **(C)** Representative macroscopic appearance of femoral condyles and tibial plateau showed joint damage (red arrow). **(D)** H&E and S&F staining of medial compartment cartilage and subchondral bone of knee joints; n = 5 (scale bar: 200 μm). **(E)** H&E staining of synovial membrane of the medial compartment; n = 5 (scale bar: 100 μm). **(F–H)** Quantitative analysis of cartilage degeneration score, subchondral bone damage score and synovitis inflammation score in three groups, n = 5. All data represent mean ± S.E.M.*p < 0.05, **p < 0.01, ***p < 0.001 and ****p < 0.0001.

The male Sprague-Dawley (SD) rats (6-week-old) were acquired from the Animal Center of Nanjing Medical University (Jiangsu, China). The rats were randomized into three groups (n = 18): sham operation group, OA group, and OA with celastrol treatment group. Rats in OA and celastrol treatment groups received “medial collateral ligament transection (MCLT) + partial meniscectomy (pMMT)” and rats in sham operation group underwent sham surgery. After surgery, OA rats in the celastrol treatment group were injected with 1 mg/kg celastrol (Sigma-Aldrich, Darmstadt, Germany) intra-articularly per week, and rats in the OA group were injected with normal saline. This treatment started 8 weeks after surgery and continued for 8 weeks until the rats were sacrificed (Figure 1A).

### OA severity assessment

The maximum length of coronal plane of rat knee joint was taken as the diameter of the knee joint, and measured with vernier caliper (the distance from the medial condyle of the femur to the lateral condyle plus the thickness of swollen joint synovium).

### Micro-computed tomography

The knee joints of rat were harvested and fixed in 4% PFA at 8 weeks post-injections. The microstructure of the joints was analyzed by micro-CT scanner (mCT80; Scanco Medical AG). The voltage of the scanner was set as 70 kV, the current was 114 μA, and the resolution was 15.6 μm per pixel. 3D reconstruction images were obtained using Scanco Medical software. Total bone volume (BV), total bone volume/total tissue volume (BV/TV), trabecular number (TB.N), thickness (TB.TH) and separation (TB.SP) were also analyzed. Table 1.

**TABLE 1:** The primers of genes.

Name	Primer list
rTLR2-F	5′- TCA​CTG​TTC​TCC​AAT​CTC​ACA​A -3′
rTLR2-R	5′-CAG​CCC​AGC​AAA​ATC​TAT​TCT​C -3′
rNFKB-F	5′-GTT​CGA​TGC​TGA​TGA​AGA​CTT​G -3′
rNFKB-R	5′-AAA​CTC​TGA​GTT​GTC​GAC​AGA​T -3′
rMMP13-F	5′- AGC​TCC​AAA​GGC​TAC​AAC​TTA​T-3′
rMMP13-R	5′- GTC​TTC​ATC​TCC​TGG​ACC​ATA​G-3′
rCOX2-F	5′- ATC​AGA​ACC​GCA​TTG​CCT​CT-3′
rCOX2-R	5′- GCC​AGC​AAT​CTG​TCT​GGT​GA-3′
rGAPDH-F	5′- AAC​TCC​CAT​TCT​TCC​ACC​TTT​G-3′
rGAPDH-R	5′- CTC​TTG​CTC​TCA​GTA​TCC​TTG​C-3′
hTLR2-F	5′-CTT​CAC​TCA​GGA​GCA​GCA​AGC​A-3′
hTLR2-R	5′-ACA​CCA​GTG​CTG​TCC​TGT​GAC​A-3′
hGAPDH-F	5′-TCA​GTG​GTG​GAC​CTG​ACC​TG-3′
hGAPDH-R	5′-TGC​TGT​AGC​CAA​ATT​CGT​TG-3′

### Molecular modeling

All procedures were performed with Maestro (version 9.0.111, Schrödinger, LLC, New York, NY). The structure of celastrol was optimized to predict its protonation state and form stereoisomers. The protein structure of TLR2 was obtained from https://www.rcsb.org/(PDB DOI: 10.2210/pdb 1FYW/pdb). Protein Preparation Wizard was used to remove water molecules and heteroatom groups and add hydrogen. The grid-enclosing box was placed on the celastrol’s docking region, and the glide module was used to dock the compounds using the default parameters.

### Cell culture

Rat cartilages were isolated from the knee, shoulder, elbow and femur of newborn SD rats (within 24 h). Human cartilages were collected from OA patients (51–64 years old; Kellgren–Lawrence grade IV; n = 4) during total knee replacement surgeries. For primary chondrocyte culture, fresh cartilages were washed with phosphate-buffered saline (PBS) under sterile conditions. Then, the cartilages were cut into 1 mm^3^ pieces and digested with 0.2% collagenase II in DMEM/F12 at 37°C overnight. After filtration, the chondrocytes were seeded and cultured in culturing dishes at 37°C and 5% CO_2_. Primary chondrocytes were identified by immunofluorescence (Supplementary Figure S1).

### Cell viability assay

In order to evaluate the cytotoxicity of celastrol to chondrocytes, the cell counting kit -8 (CCK-8) assay (Dojindo Co., Kumamoto, Japan) was used according to manufacturer’s protocol. The same density of rat chondrocytes (rCHs) was seeded in 96-well plate and then treated with celastrol (0.1–2 μM) for 24 h. After washed with PBS, the rCHs in each well were incubated with 10% CCK-8 solution at 37°C for 2 h. The absorbance was measured at 450 nm by a microplate reader (Thermo Scientific, Logan, UT, USA).

### Quantitative real-time polymerase chain reaction

Total RNA was extracted from rCHs using TRIzol reagents (Vazyme, China). The HiScript Reverse Transcriptase Kit (Vazyme, China) was used to convert RNA into cDNA. PCR was performed with standard procedure. The primers used are listed in Table 1.

### Western blot analysis

RIPA lysis buffer with 1 mM protein phosphatase inhibitor was added into rCHs for protein extraction. The lysate was collected and centrifuged for 10 min at 12,000 r/min at 4°C. A BCA protein assay kit (Thermo Scientific) was used to determine the concentration of protein. The proteins were separated by 10% SDS-polyacrylamide gels, and then transferred onto PVDF membranes (Bio-Rad, Hercules, CA, USA). The block solution was the TBS (Tris-HCL Buffer Solution) containing bovine serum albumin (0.5%) (BSA, Sigma-Aldrich, Darmstadt, Germany) and Tween-20 (0.1%) (Bio Froxx, Guangzhou, China). The membranes were blocked with block solution for 1 h at room temperature, incubated with antibodies against matrix metalloproteinase 13 (MMP13, 1:1000, GTX100665, GeneTex, CA, USA), CollagenII (1:1000, ab34712, Abcam, Cambridge, UK), cyclooxygenase-2 (COX2, 1:1000, #12282, Cell Signaling Technology, Danvers, USA), TLR2 (1:1000, #29071, SAB, Nanjing, China), p-NF-κB (1:1000, #3033T, Cell Signaling Technology, Danvers, USA), NF-κB (1:1000, #8242T, Cell Signaling Technology, Danvers, USA) at 4°C overnight. Next, the TBST was used to wash membranes and the membranes incubated with horseradish peroxidase-conjugated secondary antibodies for 1 h. The ChemiDocXRS + Imaging System (Tanon, Shanghai, China) was used to detect the signals. The protein bands were quantitatively analyzed by ImageJ.

### Histopathologic analysis

The affected knee joints were collected after the SD rats were sacrificed. The knee joints were fixed in 4% PFA for 24 h and decalcified with 10% EDTA for 2 months. After decalcification, the knee joints were embedded in paraffin and continuously sectioned at 5 μm. The tissue sections were stained with safranin O-fast green (S&F) and hematoxylin and eosin (H&E). Cartilage degeneration and subchondral bone damage were evaluated according to the international scoring system of the OA Research Association, and the severity of synovitis was graded using a scoring system as previously described (Gerwin et al., 2010).

### Enzyme linked immunosorbent assay

Chondrocytes were seeded in 12-well plates, and supernatants were collected after IL-1β treatment with or without celastrol for 24 h. According to the manufacturer’s protocol, levels of IL-6 and PGE2 were estimated using commercial ELISA kit (Multi Sciences, Hangzhou, China), with OD values measured at 450 and 570 nm.

### Immunohistochemical staining

The tissue sections were dewaxed in xylene solution, and then 3% hydrogen peroxide was used to inhibit the activity of endogenous peroxidase. The slices were incubated with 0.4% pepsin (Sigma-Aldrich) in 1 mM hydrochloric acid at 37°C for 1 h for antigen repair. The block solution was the PBS solution containing 5% BSA. After blocking with block solution at 37°C for 30 min, the slices were incubated with the primary antibodies against MMP13 (1:1000, GTX100665, GeneTex, CA, USA), CollagenII (1:1000, ab34712, Abcam, Cambridge, UK), TLR2 (1:1000, #29071, SAB, Nanjing, China) at 4°C overnight, and finally with a HRP-conjugated secondary antibody at room temperature for 1 h. Then, the slices were washed with PBS and developed, and sealed after the re-staining with methyl green (0.2%). The positive cells were counted by ImageJ.

### Cell immunofluorescence

The cells were seed in 48-well plates. After IL-1β (10 ng/ml) treatment with or without celastrol (0.025, 0.0.05 and 0.1 μM) for 24 h, the cells were washed with PBS, fixed in 4% PFA for 20 min, and permeabilized with 0.4%Triton-100X for 10 min. The block solution was the PBS solution containing BSA (0.5%) and Tween-20 (0.1%). After blocking with block solution at room temperature for 1 h, the cells were incubated with primary antibody at 4°C overnight and washed with PBS. Then the cells were incubated with FITC-conjugated secondary antibodies at room temperature for 1 h, stained with Hoechst33258 for 10 min, and observed with a microscope. The average fluorescence intensity was measured by ImageJ.

### Statistical analysis

All data are expressed as the Means ± Sem. Statistical significance between multiple groups was determined by one-way analysis of variance (ANOVA) and Tukey test was conducted post hoc test using GraphPad Prism 8. T-test was used to analyze statistical difference between two independent groups. Differences were considered statistically significant when *p* < 0.05.

## Results

### Celastrol alleviated the progression of OA in rat model

We performed surgery to remove the medial meniscus from the right knee of 6-week-old rats to induce OA progression. Eight weeks after surgery, the rats received right articular joint injections of saline or 1 mg/kg celastrol once a week for 8 weeks (Figure 1A). We measured the 5-min walking distance and times reared within 5 min at Week 16, and found that celastrol improved the walking ability of rats with OA (Supplementary Figures S2A,B). Then, we observed the lateral view of affected limbs in general, and found that the right knee joint stiffness in full extension was improved in OA group after celastrol treatment (Supplementary Figure S2C). Moreover, we assessed the degree of joint swelling by the maximal coronal diameter of the right knee joint with vernier caliper at week 16, and found that celastrol ameliorated joint swelling compared to OA group (Figure 1B, Supplementary Figure S2D). In addition, celastrol group showed a smooth surface in the posterior portion of the medial tibial plateau and medial femoral condyle, while severe rough surface was observed in OA group (red arrows), suggesting celastrol could alleviate bone wear (Figure 1C). H&E and S&F staining performed in joint section displayed significant damage to the cartilage surfaces, loss of proteoglycans, and thinning articular in OA group, and improved cartilage integrity in celastrol group (Figure 1D). Blinded OARSI scoring was used to measure the severity of OA, and celastrol group had significantly lower cartilage degeneration score and subchondral bone damage score compared to OA group (Figures 1F,G). Moreover, H&E staining and synovial membrane inflammation score indicated the presence of synovial hypercellularity and thickening in the OA group, whereas celastrol treatment alleviated synovitis (Figures 1E,H).

### Celastrol reduced osteophyte formation and bone resorption induced by “MCLT+pMMT” *in vivo*

**FIGURE 2:**
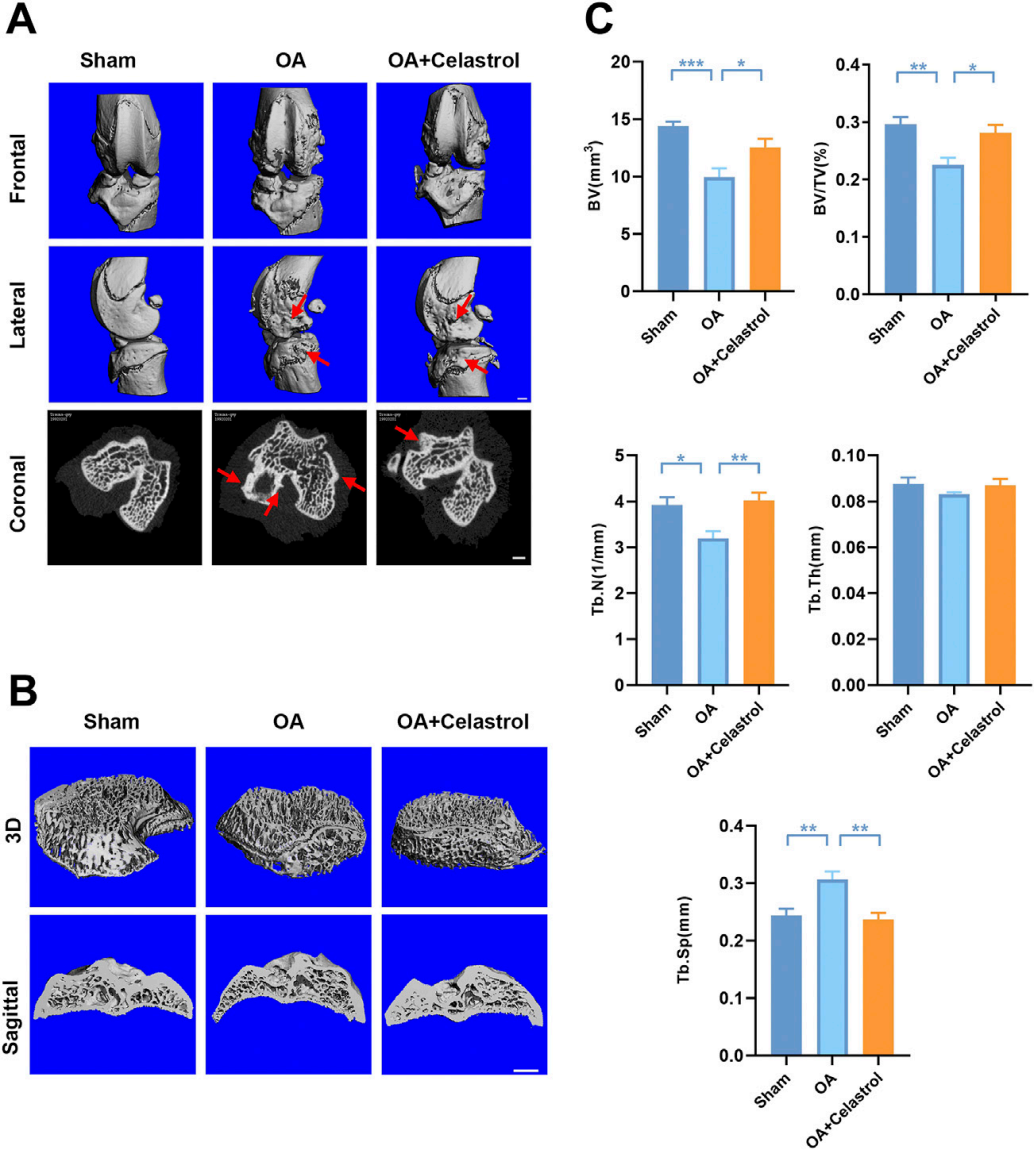
Celastrol reduced osteophyte formation and bone resorption induced by MCLT+pMMT *in vivo*. **(A)** Representative μCT images of frontal, lateral and coronal views of the knee joints from different groups (scale bar: 1 mm). **(B)** Representative three-dimensional μCT images of subchondral bone and sagittal views of medial compartment subchondral bone (scale bar: 1 mm). **(C)** Quantitative analysis of BV, BV/TV, Tb.N Tb.Th and Tb. Sp, n = 6. All data represent mean ± S.E.M. *p < 0.05, **p < 0.01 and ***p < 0.001.

In OA joints, osteophyte formation developed as a characteristic structural change of OA, which was found in micro-computed tomography (μCT) analysis after “MCLT+pMMT” treatment. Remarkably, celastrol group presented less osteophyte (red arrows) formation compared to OA group (Figure 2A). Meanwhile, subchondral bone resorption followed by increased bone remodeling played an important role in the pathogenesis of OA. To investigate the effects of celastrol on the subchondral bone remodeling of OA, we reconstructed the tibian plateau subchondral bone and analyzed the microstructure parameters. “MCLT+pMMT” significantly induced bone resorption, as shown by the increased osteolysis in subchondral bone of the tibia. And celastrol treatment reduced bone loss and increased bone mass in subchondral bone (Figure 2B). BV of tibial plateau subchondral bone was significantly lower in the OA group than the sham and celastrol groups (Figure 2C). In addition, BV/TV and Tb.N were decreased, and Tb. Sp was increased in OA group, which strongly revealed the suppressed bone destruction effect of celastrol.

### Celastrol inhibited IL-1β-induced inflammatory mediators in rat chondrocytes

**FIGURE 3:**
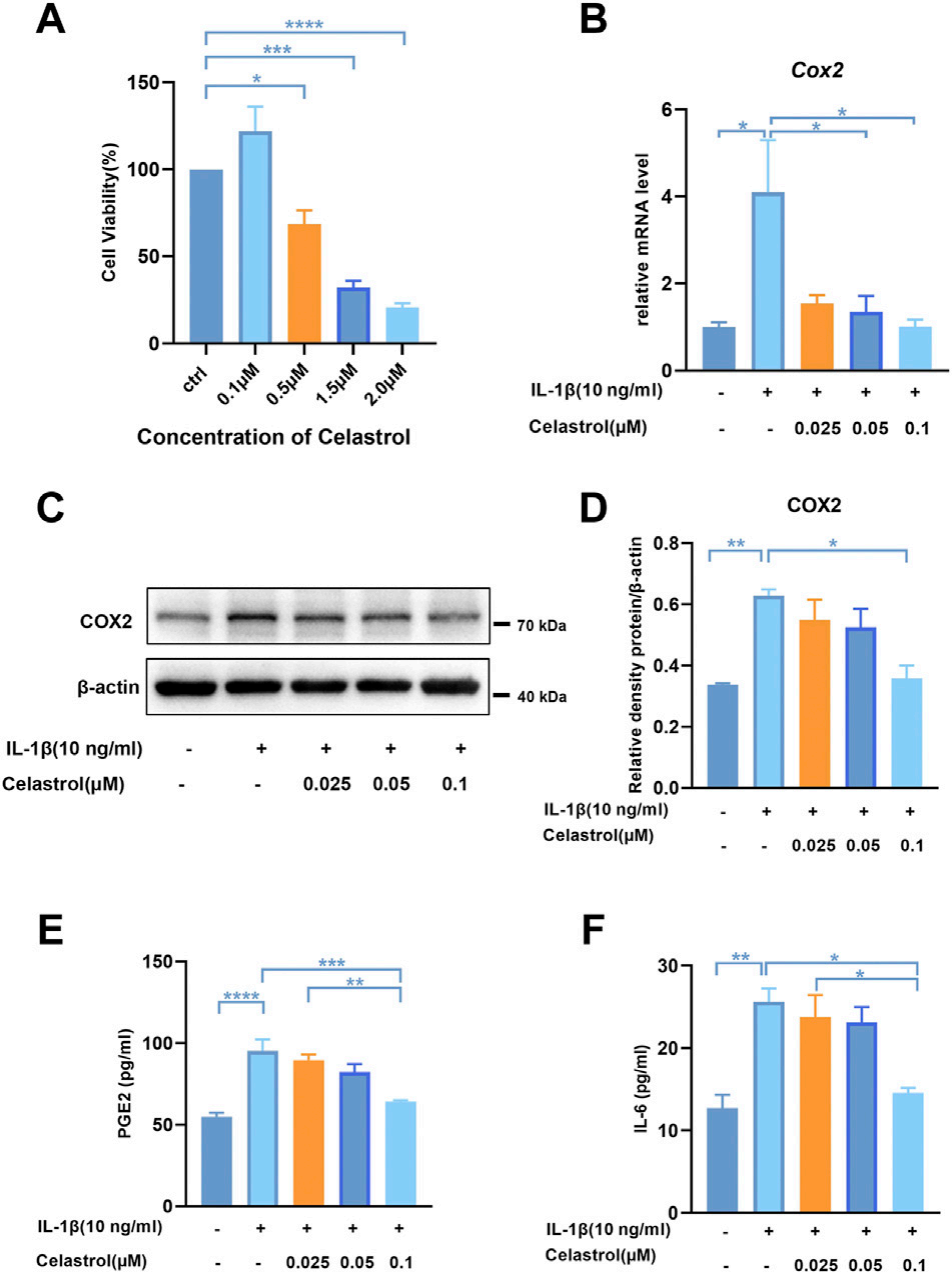
Celastrol inhibited IL-1β-induced inflammatory mediators in rat chondrocytes. **(A)** Rat chondrocytes viability for 24 h treated with celastrol at different concentrations examined by CCK-8 kit. **(B)** The mRNA levels of COX2 in rat chondrocytes were assayed by real-time PCR. **(C)** Representative Western blot results for COX2 in rat chondrocytes treated as indicated. **(D)** Quantification of Western blot data from **(C)**. **(E,F)** Levels of PGE2 and IL-6 were assayed by ELISA. All data represent mean ± S.E.M.*p < 0.05, **p < 0.01, ***p < 0.001 and ****p < 0.0001. All experiments were repeated 3 times.

To assess the cytotoxicity of celastrol on chondrocytes, we treated rCHs with different concentrations (0.1–2 μM) of celastrol for 24 h. Celastrol treatment (0.1 μM, 24 h) had no significant cytotoxic effect on rCHs and even slightly promoted the proliferation of rCHs. However, high concentrations (0.5, 1.5 and 2 μM) of celastrol treatment had a dose-dependent cytotoxic effect in terms of cell viability (Figure 3A). Referring to CCK8 analysis, celastrol was divided into low, medium and high dose groups according to different concentrations (0.025, 0.05, 0.1 μM). We examined IL-1β-induced inflammatory cytokines and found that the increased COX2 expression and PGE2 levels were significantly alleviated after celastrol treatment (Figures 3B–E). Besides, elevated IL-6 levels induced by IL-1β were also decreased significantly with the treatment of celastrol (Figure 3F).

### Celastrol inhibited matrix degradation in rat chondrocytes

**FIGURE 4:**
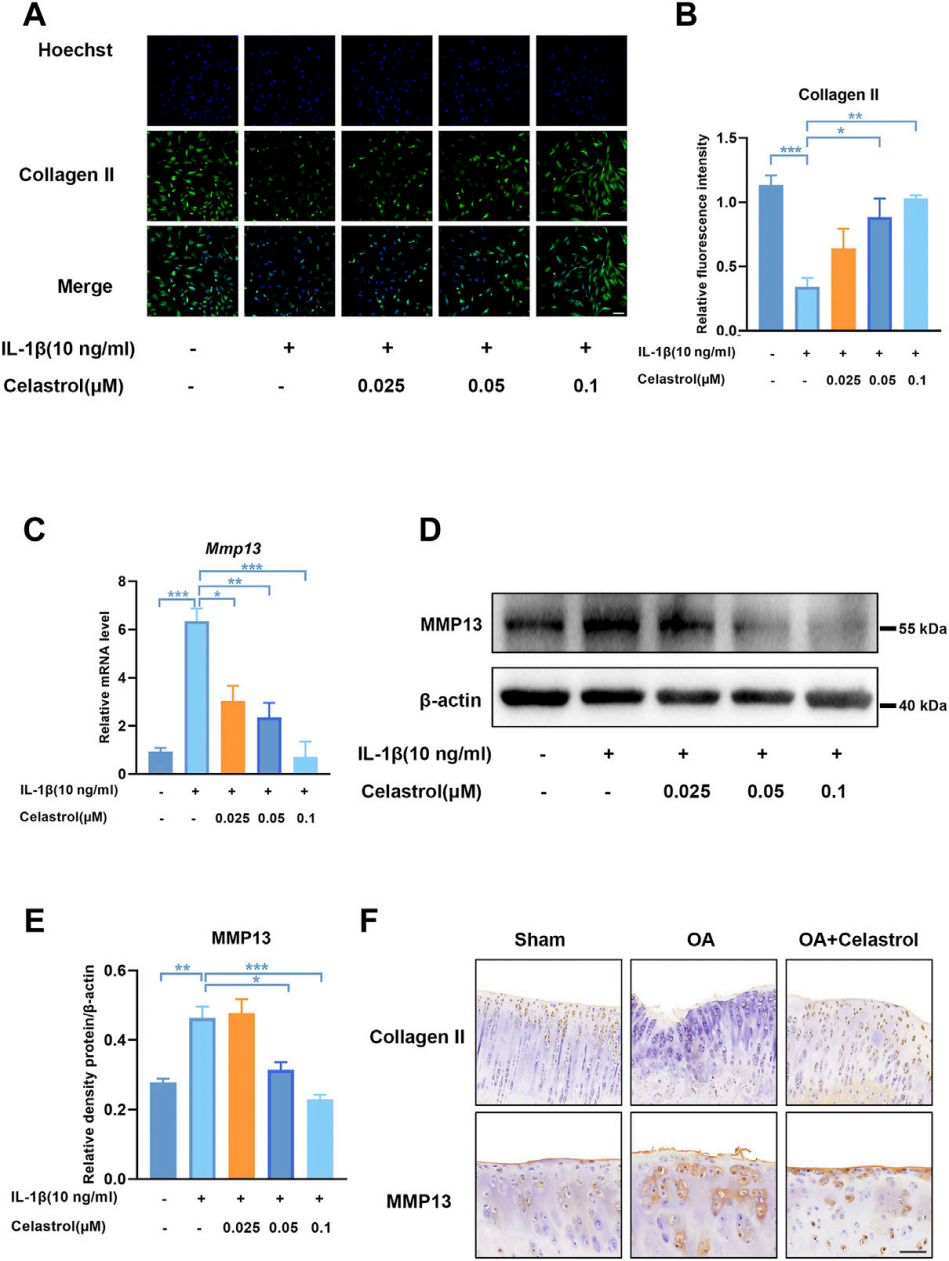
Celastrol inhibited matrix degradation in rat chondrocytes. **(A)** Representative immunofluorescence staining for Collagen Ⅱ in rat chondrocytes (green: Collagen Ⅱ, blue: DAPI, scale bar:100 μm). **(B)** Quantification of the immunohistochemical staining data from A. **(C)** The mRNA levels of MMP13 in rCHs were assayed by real-time PCR. **(D)** Representative Western blot results for MMP13 in rCHs treated as indicated. **(E)** Quantification of Western blot data from D. **(F)** Immunohistochemical staining for CollagenⅡ and MMP13 in the affected joint cartilage of rats from different groups (scale bar:50 μm). All data represent mean ± S.E.M.*p < 0.05, **p < 0.01 and ***p < 0.001. All experiments were repeated 3 times.

As Collagen II was the major ECM component, IL-1β inhibited the synthesis of matrix collagen and induced downregulation of COL2A1 in IL-1β group. Fluorescence analysis showed a markedly higher density of Collagen II in rCHs in “IL-1β+celastrol group” than in IL-1β group with a celastrol dose-dependant manner (Figures 4A,B). OA affected joints are mainly characterized by the degradation of cartilage extracellular matrix, and especially matrix metalloproteinase13 (MMP13) is the most effective collagen II degrading enzyme. The Western blot and real-time PCR analysis of rCHs stimulated with IL-1β showed that the expression of MMP13 was significantly decreased by celastrol treatment in a dose-dependent manner (Figures 4C–E). Similar to the results *in vitro*, immunohistochemical staining of rat cartilage with OA showed that the expression of MMP13 was significantly rescued by celastrol treatment. Moreover, the immunohistochemistry revealed the loss of Collagen II was alleviated by celastrol treatment (Figure 4F). Altogether, celastrol promoted ECM anabolism via down-regulating the expression of catabolic factors and affecting Collagen II deposition in pathological chondrocytes.

### The expression of TLR2 was up-regulated in OA patients and *in vitro* model of human chondrocytes

**FIGURE 5:**
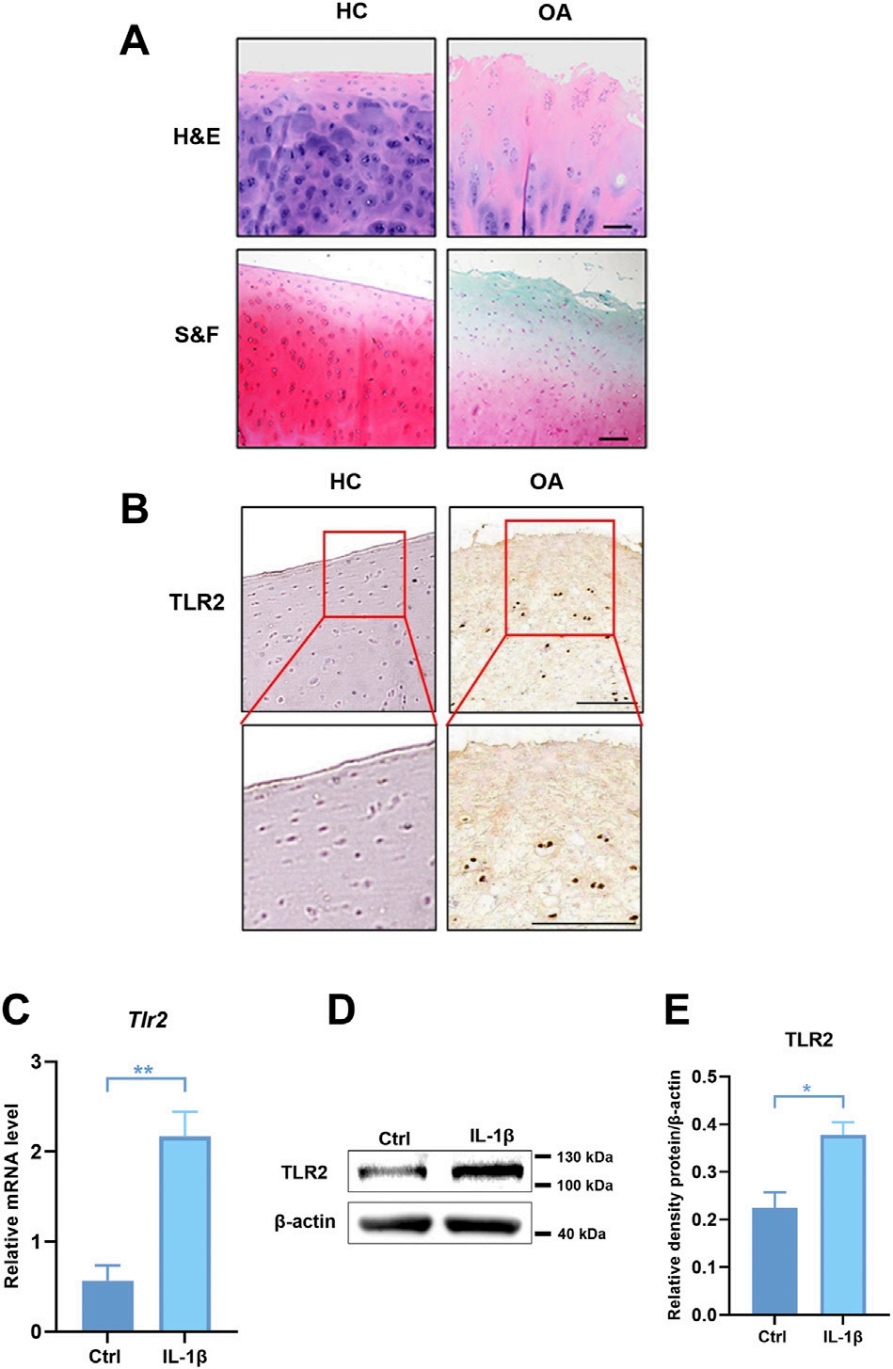
The expression of TLR2 was up-regulated in OA patients and in human chondrocytes treated with IL-1β. **(A)** H&E and S&F staining of healthy control and OA patients (scale bar: 200 μm). **(B)** Immunohistochemical staining for TLR2 in healthy control and human OA samples. **(C)** The mRNA levels of TLR2 in hCHs treated with or without IL-1β (10 ng/ml) were assayed by real-time PCR. **(D)** Representative Western blot results for TLR2 in hCHs treated with or without IL-1β (10 ng/ml). **(E)** Quantification of Western blot data from **(D)**. All data represent mean ± S.E.M.*p < 0.05, **p < 0.01. All experiments were repeated 3 times.

As the pathogenesis of OA is associated with abnormal TLRs-mediated innate immune-inflammatory responses, we analyzed the expression of TLRs in degenerative meniscus cells of OA patients (Supplementary Figure S3) (Jiang et al., 2021). Among these, the expression of TLR2 and TLR4 was much higher than the others, especially TLR2. We further collected cartilage from OA patients and healthy cartilage to evaluate cartilage wear and TLR2 expression. H&E and S&F staining showed that the cartilage surface of OA patients was seriously damaged. Chondrocytes were disorderly arranged, and the tide line was distorted (Figure 5A). Compared with healthy control, the expression of TLR2 increased in OA patients (Figure 5B). To further verify these results, we stimulated human chondrocytes (hCHs) with IL-1β to establish a model of OA *in vitro*. We validated that the mRNA level of TLR2 was significantly up-regulated *in vitro* model (Figure 5C). Moreover, we found that the protein level of TLR2 also significantly increased (Figures 5D,E).

### Celastrol inhibited TLR2-triggered signals in rat chondrocytes

**FIGURE 6:**
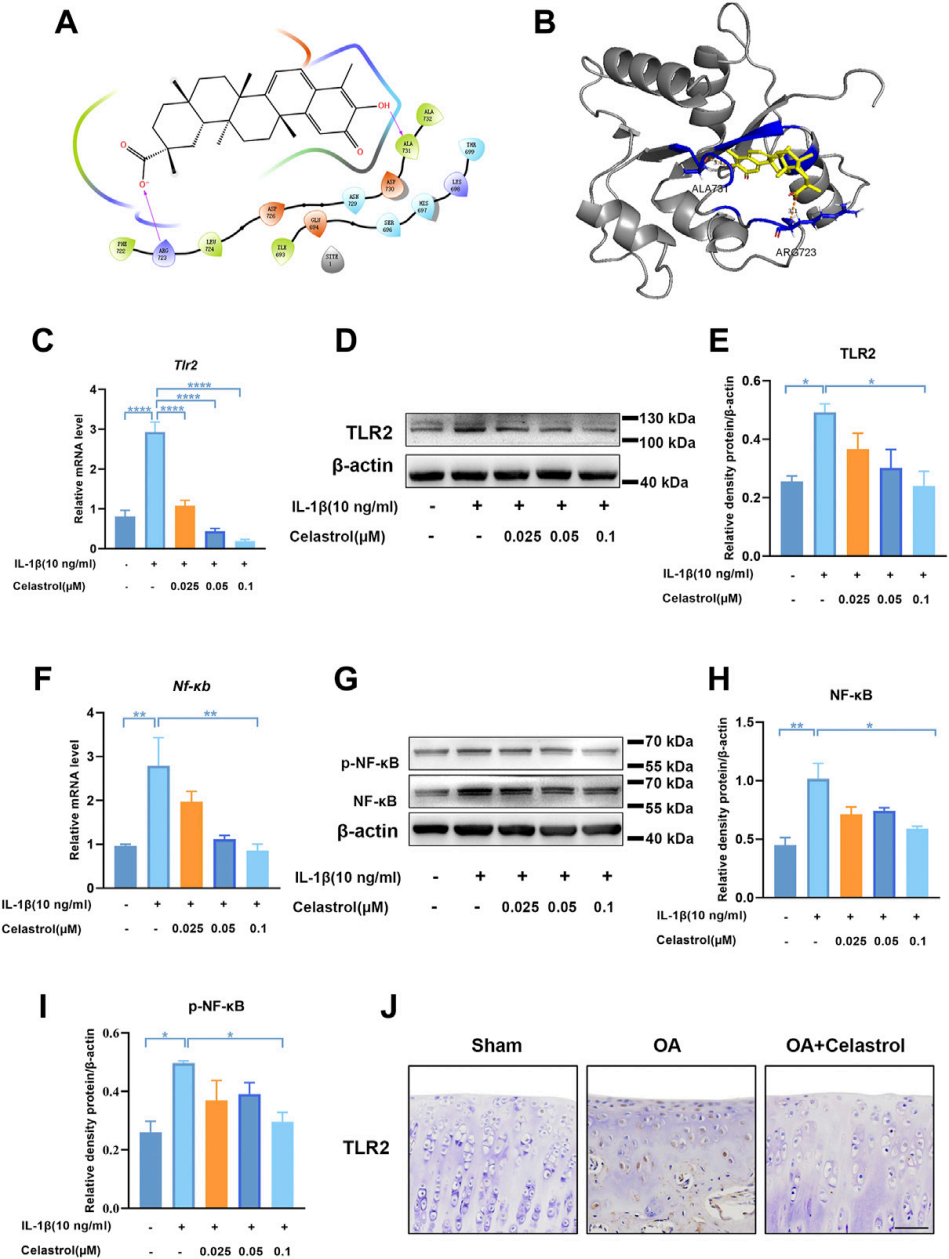
Celastrol inhibited TLR2-triggered signals in rat chondrocytes. **(A,B)** Interaction diagrams of celastrol docked to TLR2 using Maestro software. **(C,F)** The mRNA levels of TLR2 and NF-κB in rat chondrocytes were assayed by real-time PCR. **(D,G)** Western blot results for TLR2, p-NF-κB and NF-κB in rat chondrocytes with or without celastrol. **(E,H,I)** Quantification of Western blot data from D and G. **(J)** Immunohistochemical staining for TLR2 in the affected joint cartilage of rats from different groups (scale bar: 50 μm). All data represent mean ± S.E.M. *p < 0.05, **p < 0.01, ***p < 0.001 and ****p < 0.0001. All experiments were repeated 3 times.

Docking simulation analysis of celastrol docked to TLR2 by maestro software showed that TLR2 could interact with celastrol (Figures 6A,B). To further investigate the effect of celastrol on TLR2-triggered signals, we first measured the TLR2 expression of rCHs with IL-1β stimulation (Figures 6C–E). As we can see TLR2 expression significantly increased under IL-1β stimulation, and decreased after celastrol treatment. With the activation of the TLR2 signaling pathway, the downstream NF-κB and NF-κB phosphorylation was subsequently induced to activate. Western blot and real-time PCR analysis showed that the IL-1β-stimulated increasement in NF-κB and NF-κB phosphorylation expression was significantly inhibited after celastrol treatment in rCHs (Figures 6F–I). *In vivo*, compared with OA group we also found the decrease in TLR2 expression in the “OA+celastrol” group in the cartilage (Figure 6J). Thus, we concluded that celastrol attenuated inflammation and matrix degradation via regulating TLR2-dependent pathway.

## Discussion

For a long time, OA was known as a degenerative disease of joints, characterized by articular cartilage degeneration, ECM degradation, bone loss and synovitis. (Bultink and Lems, 2013). Recently, OA has been considered a multifactorial disease rather than a degenerative one, in which low-grade, chronic inflammation plays a crucial role. (Robinson et al., 2016). Previous studies suggested that low-grade inflammation mediated by innate immune system, especially TLRs, contributed to cartilage damage and osteoporosis during OA development (Miller et al., 2019; Saxena et al., 2021). Despite many studies that attempted to delay cartilage degeneration, no effective treatment has yet focused on the roles of innate immunity (Hochberg et al., 2012). Our work demonstrate that celastrol can improve various pathological changes of OA by inhibiting innate immunity and provide an “entry point” for treatment.

Recent studies have revealed that TLRs are closely related to OA. Stimulation of TLRs ultimately activated transcription factors such as NF-κB and activator protein 1 (AP1), leading to initiate an inflammatory transcription program (Scanzello et al., 2008). Among all the functional TLRs in human, TLR1-TLR7 and TLR9 have been detected in the synovium of OA patients (Scanzello and Goldring, 2012), and the up regulation of TLR2 and TLR4 can be found in human knee lesions (Liu-Bryan, 2013). In the innate immune response, human articular chondrocytes express TLRs. Among all TLRs, TLR2 and TLR4 are the main regulators of innate immunity activated in OA (Barreto et al., 2017; Hamasaki et al., 2020; Korotkyi et al., 2020; Korotkyi et al., 2021). Compared to TLR4, TLR2 plays a crucial role in the early stage of innate immune response, and the effect of controlling the initial inflammatory response in the joint cavity is critical for the prognosis of OA (Abdollahi-Roodsaz et al., 2008; Wojdasiewicz et al., 2014). In this study, we focused on TLR2 and confirmed that the expression of TLR2 was up-regulated in chondrocytes of both OA patients and OA model rats. Our results suggest that cartilage protection can be achieved by inhibiting TLR2. In addition inhibition of TLR2 may suppress osteoclastic bone resorption, thereby improving osteoporosis (Wallimann et al., 2021). NF-κB is a crucial regulator of innate immunity (Chen et al., 2018) and is considered to be an important regulator of TLR induced response (Carmody and Chen, 2007). Aberrant NF-κB regulation is involved in the development of OA and OP(Chen et al., 2020). Therefore, inhibition of the TLR2/NF-κB signaling pathway may be critical to the treatment of OA.

Celastrol is widely used to treat autoimmune and inflammatory diseases such as rheumatoid arthritis (RA), systemic lupus erythematosus, and nephropathy. As a quinone methyl triterpene, celastrol was extracted from the root of Trypterygium wilfordii, which has attracted extensive attention because of its anti-inflammatory and anti-cancer effects (Sethi et al., 2007; Kim et al., 2009). Recent studies validated that Tripterygium wilfordii (TW) and its extracts have regulatory effects on innate immune cells including macrophages and neutrophils, as well as a variety of innate immune molecules including cytokines, adhesion molecules, PRR and complement molecules (Li et al., 2019). A related study found that celastrol could promote autophagy *in vitro* and *in vivo* to protect chondrocyte (Feng et al., 2020). Although celatrol has a powerful anti-inflammatory effect, few studies have examined whether such effects are brought out by inhibiting innate immunity, especially TLR2, which has only been reported in multiple sclerosis and RA (Abdin and Hasby, 2014; Lu et al., 2021). Furthermore, whether celastrol can regulate TLRs-mediated innate immunity in OA has never been investigated to the best of our knowledge. Moreover, a growing number of studies have observed numerous adverse effects associated with oral TW, especially hepatotoxicity, which has hindered its widespread use in clinical practice (Song et al., 2020). As for Tripterygium wilfordii polyglycosides tablet, its efficacy and toxicity are difficult to depict because of its complex components, thus greatly limiting its clinical application. Therefore, in order to reduce drug side effects, we adopted intra-articular injection to observe whether local administration of celastrol could inhibit the innate immunity and delay the progression of OA.

Our results showed that celastrol not only significantly inhibited the expression of inflammatory factors such as COX2, IL-6, and PEG2 but also suppressed the expression of MMP13 and degradation of CollagenⅡ *in vitro* **.** *In vivo* the results showed that the joint wear and tear was significantly reduced in celastrol treated group compared with OA group. All these results suggest that celastrol has an excellent protective effect on the cartilage of OA in rats. In addition, our study showed the inhibitory effect of celastrol on bone resorption. Knee joints in OA group showed significant bone loss based on a significant decrease in bone volume and BV/TV, a decrease in Tb.N, and an increase in Tb. Sp. Therefore, our therapeutic goal is not simply to relieve pain, but more importantly to inhibit articular cartilage destruction and osteoporosis of subchondral bone. All above results indicate that celastrol can protect cartilage and inhibit OP of subchondral bone, which makes it a potential drug for OA treatment.

IL-1β is a vital pro-inflammatory factor in the development of OA, which can induce chondrocyte inflammation and catabolism independently, and can also act on articular cartilage and other components in combination with other mediators. As a damage factor, IL-1β induces the release of inflammatory mediators (Kobayashi et al., 2005). These inflammatory mediators aggravate cartilage destruction by activating MMPs and inhibiting the synthesis of collagen and glycoaminoglycan (GAG) (Scanzello et al., 2008; Nair et al., 2012; Gomez et al., 2015). Therefore, IL-1β is often used to establish OA models *in vitro* (Dunn et al., 2014; Wang et al., 2021). We simulated the inflammatory microenvironment of OA *in vitro* and found that IL-1β up-regulated the expression of TLR2 and NF-κB in chondrocytes. Further results showed that celastrol inhibited IL-1β-induced upregulation of TLR2 and NF-κB in chondrocytes to reduce inflammation and extracellular matrix degradation.

**FIGURE 7:**
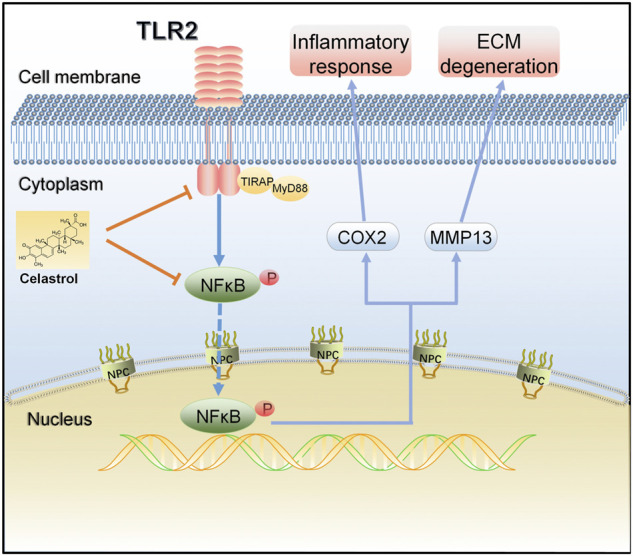
A schematic diagram of the proposed mechanism of celastrol on TLR2/NF-κB signals in rat chondrocytes. Celastrol treatment negatively regulated TLR2 activation and NF-κB phosphorylation in IL-1β-induced chondrocytes, thereby inhibiting inflammation and matrix degradation.

In summary, we demonstrated that the TLR2/NF-κB signaling pathway involved in early OA and might be a potential therapeutic target for slowing or altering the progression of OA. Celastrol showed anti-inflammatory and chondroprotective effects both *in vivo* and *in vitro* by down regulating the TLR2/NF-κB pathway, effectively inhibiting the release of inflammatory factors, cartilage degradation and bone loss, indicating that celastrol had a therapeutic effect on OA (Figure 7). Our study provides a new possible therapeutic approach for OA treatment, which could be further verified by future studies.

## Data Availability

The original contributions presented in the study are included in the article/Supplementary Material, further inquiries can be directed to the corresponding authors.
