# Supplementary figures and images for: Celastrol ameliorates osteoarthritis via regulating TLR2/NF-κB signaling pathway
Source: Front Pharmacol. 2022 Aug 10;13:963506. doi: 10.3389/fphar.2022.963506 (PMC9399520; doi:10.3389/fphar.2022.963506)

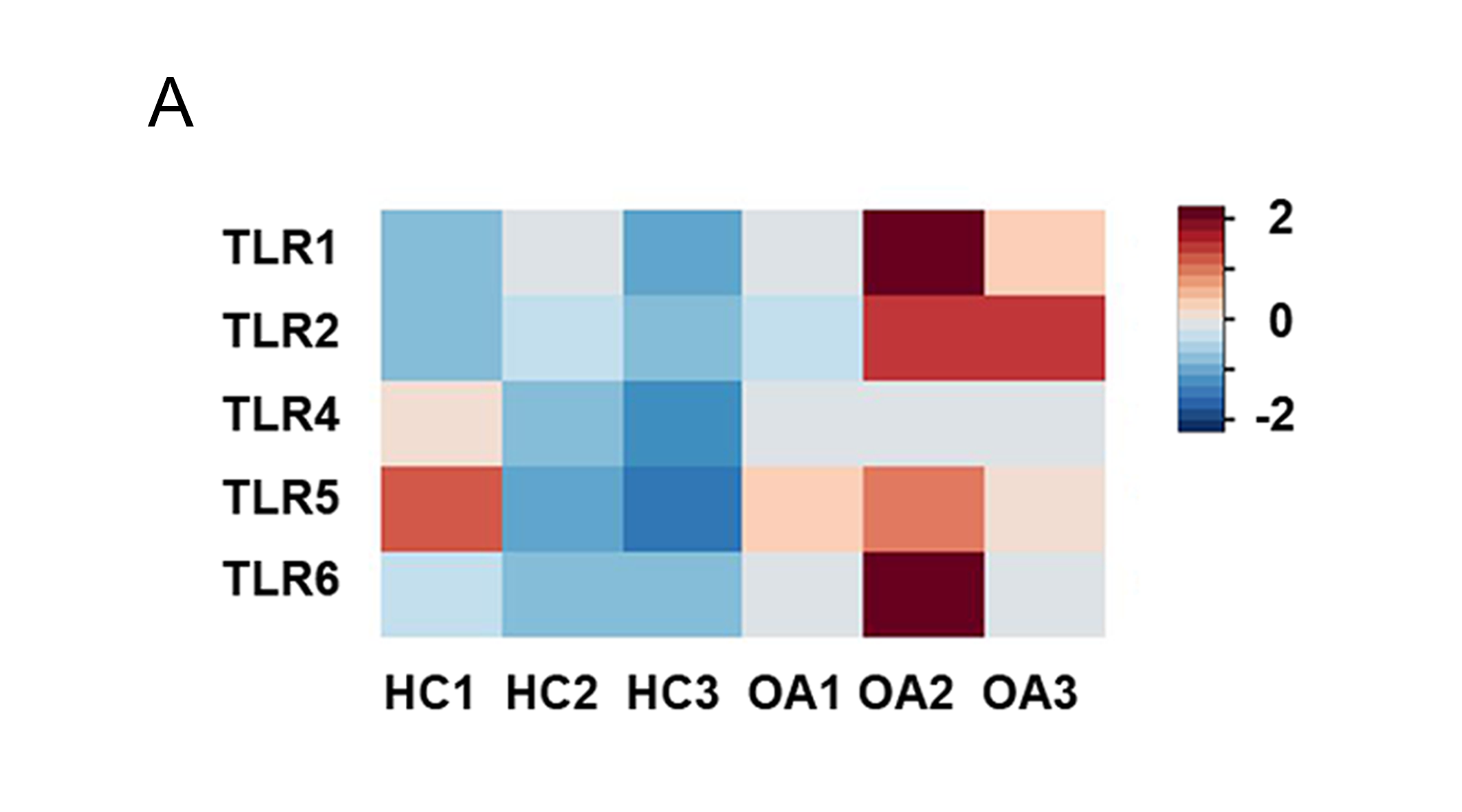

Supplement: Supplementary file 1 [file Image3.TIF]

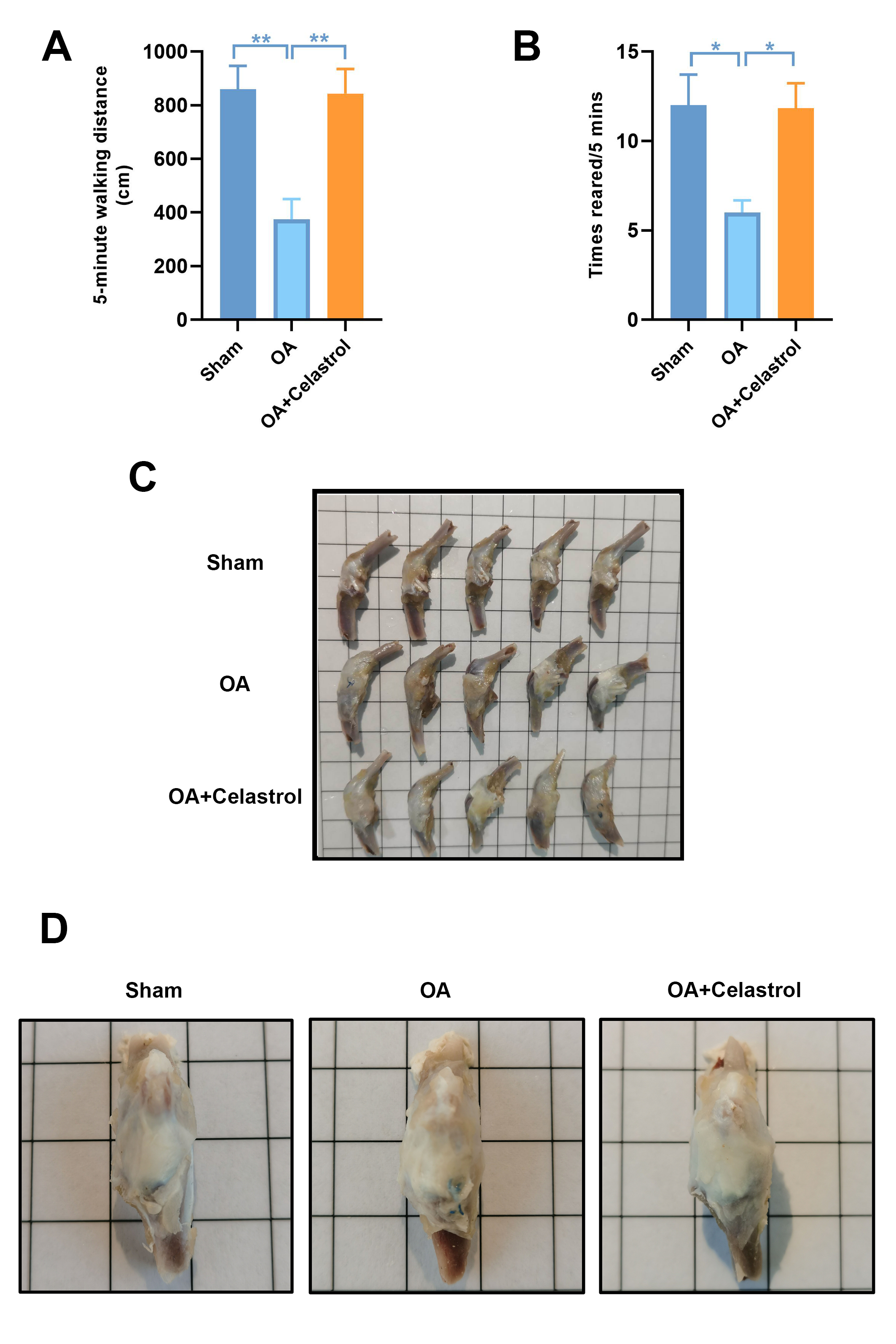

Supplement: Supplementary file 2 [file Image2.TIF]

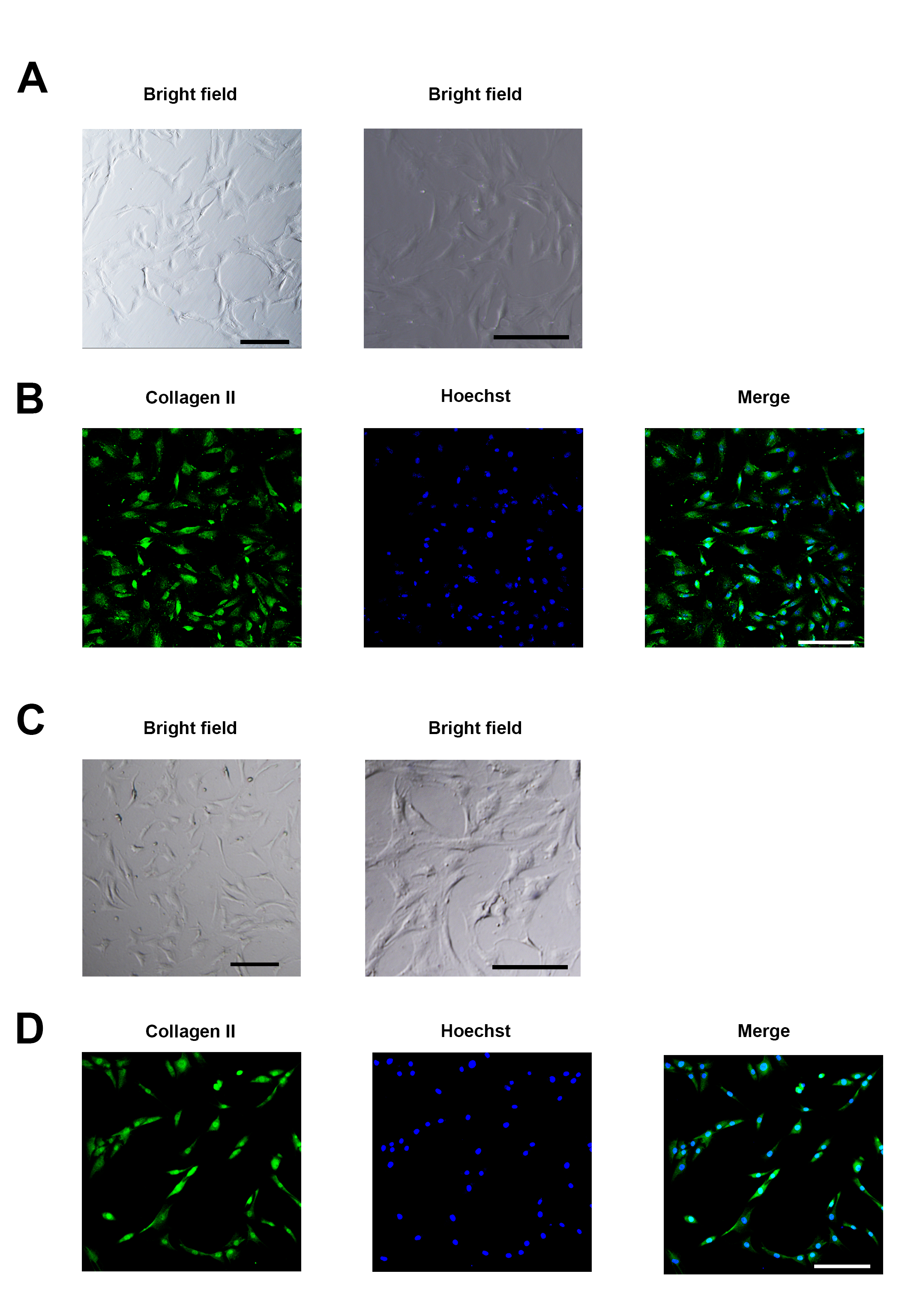

Supplement: Supplementary file 3 [file Image1.TIF]
